# Changes in androgens and insulin sensitivity indexes throughout pregnancy in women with polycystic ovary syndrome (PCOS): relationships with adverse outcomes

**DOI:** 10.1186/1757-2215-3-23

**Published:** 2010-10-13

**Authors:** Angela Falbo, Morena Rocca, Tiziana Russo, Antonietta D'Ettore, Achille Tolino, Fulvio Zullo, Francesco Orio, Stefano Palomba

**Affiliations:** 1 Department of Obstetrics & Gynecology, University "Magna Graecia" of Catanzaro, Catanzaro, Italy; 2 Department of Obstetrics & Gynecology, University "Federico II" of Naples, Naples, Italy; 3 Endocrinology, "Parthenope" University, Naples; Italy

## Abstract

**Background:**

Given the high rate of pregnancy and perinatal complications recently observed in patients with polycystic ovary syndrome (PCOS) and the lack of data on the serum variations in androgens and insulin sensitivity indexes in pregnant women with PCOS, the current study was aimed to assess these changes and their potential effect on pregnancy outcomes in a population of women with PCOS.

**Methods:**

Forty-five pregnant patients with ovulatory PCOS (PCOS group) and other 42 healthy pregnant women (control group) were studied assaying serum androgen levels and insulin sensitivity indexes throughout pregnancy serially, and recording obstetrical outcomes.

**Results:**

Serum androgen levels and insulin resistance indexes were significantly (*p *< 0.05) higher in PCOS than in control group at study entry, these differences were sustained throughout pregnancy, and their changes resulted significantly (*p *< 0.05) different between PCOS and control group. In PCOS patients, women who had a complicated pregnancy showed serum androgen levels and insulin sensitivity indexes significantly (*p *< 0.05) worse in comparison to subjects without any pregnancy and/or neonatal complications.

**Conclusions:**

PCOS patients have impaired changes in serum androgen levels and insulin sensitivity indexes during pregnancy. These alterations could be implicated in the pregnancy and neonatal complications frequently observed in women affected by PCOS.

## Background

Polycystic ovary syndrome (PCOS) is a heterogeneous disorder characterized by biochemical alteration, i.e. hyperandrogenism and insulin resistance, and ovarian impairment, resulting in chronic anovulation. The chronic anovulation is not the only factors influencing the reduced reproductive chances in PCOS patients. In fact, an increased incidence of complications throughout pregnancy was also observed in PCOS women after meta-analytic analysis [1].

Recently, we confirmed in a well selected population of PCOS patients an increased relative risk (RR) for complicated pregnancy [1.7, 95% confidence interval (CI) 1.12-2.96] with a total incidence of adverse outcomes of 31.4% [2]. In addition, the risk for adverse outcomes in PCOS resulted significantly related to ovarian dysfunction and biochemical hyperandrogenism [3].

Moreover, our previous study evaluated only baseline androgen levels in PCOS population and no relationship with insulin sensitivity indexes was investigated [3]. In addition, to the moment, no report was aimed to evaluate the dynamic of the hormonal and metabolic patterns in pregnant women with PCOS.

The current study is a parallel analysis of a larger previously published clinical report [2] aimed to study changes in androgens and insulin sensitivity indexes throughout pregnancy in PCOS patients, and their effect on pregnancy outcomes.

## Methods

The study was approved by the Institutional Review Board (IRB) of the Department of Obstetrics and Gynecology of the University "Magna Graecia" of Catanzaro. The protocol was carefully explained to each subject before entering the study and their written consent was obtained.

Between February 2003 and April 2008, subjects with ovulatory PCOS (PCOS group) and age- and body mass index (BMI)-matched healthy primigravidas were initially enrolled in a wider study protocol [2]. Ovulatory PCOS was diagnosed before pregnancy and confirmed at study entry according to the presence of polycystic ovaries (PCO) and clinical/biochemical hyperandrogenism without chronic oligo-anovulation [4], while the healthy state of the controls was determined by their medical history, physical and pelvic examination, complete blood chemistry, and transvaginal ultrasonography [2].

Age > 35 years, obesity (BMI > 30 Kg/m2), multiple pregnancies, gestational age higher than 7 weeks as assessed by crown-rump length (CRL) measurement, pre-malignancies or malignancies, medical conditions or other concurrent medical illnesses, cigarettes' smoking, drug/alcohol use, organic pelvic disease, uterine malformations, previous pelvic surgery, no compliance to our study-protocol, and current or previous (within the last six months) use of any hormonal and/or anti-diabetic and/or fertility drugs were considered as exclusion criteria for both cases and controls.

Each subject received folic acid (0.4 mg daily) and was instructed to follow usual diet and physical activity throughout the study.

Serial clinical, biochemical and ultrasonographic assessments for mother and/or fetal wellbeing monitoring were performed during the study according to our schedule [2,3].

Clinical evaluation consisted in obstetric examination, Papanicolau smear test (at study entry alone), Ferriman-Gallwey score [5] calculation, anthropometric measurements [including height, weight, BMI and waist-to-hip ratio (WHR)], heart rate (HR) and blood pressure (BP) assessments. In order to evaluate the subjects' physical activity, job, daily activities, and family history of complicated pregnancies specific questionnaires were completed by each woman.

Each subject underwent serial drawn blood samples to evaluate serum androgen levels and insulin sensitivity. All blood samples were obtained in the morning between 08.00 h and 09.00 h after an overnight fasting and resting in bed at study entry (within the 7^th ^week of gestation), and at the 12^th^, the 20^th ^and the 32^nd ^weeks of gestation. Particularly, total serum testosterone (T), androstenedione (A), dehydroepiandrosterone sulfate (DHEAS), and sex-hormone binding globulin (SHBG) were assayed. Glucose and insulin concentrations were assayed at fasting and, only at baseline, after oral glucose tolerance test (OGTT). The area under curve (AUC) for glucose and insulin, as well as the homeostasis model of assessment (HOMA) [fasting glucose (mmol/L) × fasting insulin (μU/mL)⁄22.5] [6], the fasting glucose-to-insulin ratio (GIR) (mg/10-4U) [7], and the free androgen index (FAI) [T (nmol/l)/SHBG × 100]) [8] were calculated in each subject.

All plasma hormone concentrations were measured by specific radioimmunoassay (RIA), whereas SHBG levels using an immunoradiometric assay (IRMA) [9]. Overall, intra- and inter-assay coefficients of variation (CV) were less than 10%.

Pregnancy/perinatal outcomes were evaluated in each subject as already detailed [2].

### Statistical analysis

The Kolmogorov-Smirnov statistic with a Lilliefors significance level was used for testing normality. Since our data resulted normally distributed, results were expressed as mean ± standard deviation (SD). Continuous variables were analyzed with the one-way analysis of variance (ANOVA) and ANOVA for repeated measures with Bonferroni test for the post hoc analysis.

For categorical variables, the Pearson *chi*-square test was performed; Fisher's exact test was used for the frequency tables when more than 20% of the expected values were lower than five.

Variations (Δ) in serum T, A, DHEAS, SHBG, and fasting insulin levels, and in HOMA, GIR and FAI were calculated at each follow-up in both groups for the overall population, these data were also adjusted for age and BMI and analyzed in sub-populations distinguished on the basis of the presence/absence of adverse pregnancy and neonatal outcomes.

Data were analyzed using the *per-protocol *analysis.

The level of statistical significance was set at *p *< 0.05 for all statistical analyses. The Statistics Package for Social Sciences (SPSS 14.0.1, 18 Nov 2005; SPSS Inc., Chicago, IL) was used for all calculations.

## Results

Forty-five and 42 subjects from the PCOS and control group, respectively, were included in the final analysis. In fact, part of the original study population (20 subjects for the PCOS group and 22 subjects for the control group) did not give their consent to participate to the current study protocol, while in 8 and 9 subjects from the PCOS and control groups, respectively, data of each follow-up visit were not available for the analysis.

**Figure 1:**
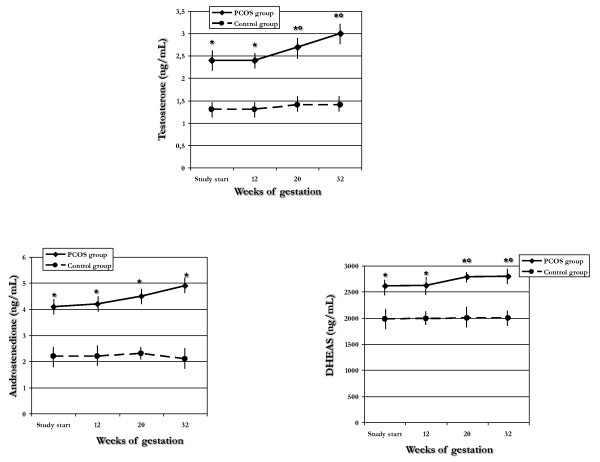
**Serum androgen levels recorded at different times throughout pregnancy in PCOS and control groups**. *p < 0.05 *vs*. control group; °*p *< 0.05 *vs*. baseline and 12^th ^gestational week.

**Figure 2:**
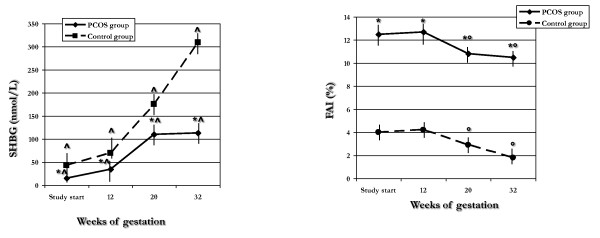
**SHBG and FAI recorded at different times throughout pregnancy in PCOS and control groups**. *p < 0.05 *vs*. control group; ^*p *< 0.05 *vs*. previous follow-up; °*p *< 0.05 *vs*. baseline and 12^th ^gestational week.

**Figure 3:**
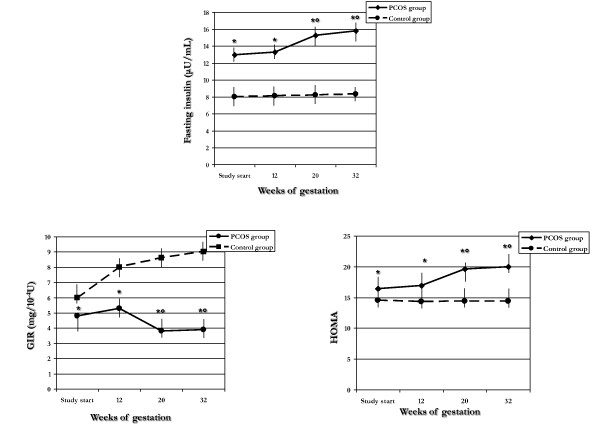
**Insulin sensitivity indexes at different times throughout pregnancy in PCOS and control groups**. *p < 0.05 *vs*. control group; °*p *< 0.05 *vs*. baseline and 12^th ^gestational week.

The main clinical data from PCOS and control group at study entry are shown in Table 1. The WHR and the Ferriman-Gallwey score, such as the AUC_glucose_, AUC_insulin_, and AUC_glucose_/AUC_insulin _were significantly (*p *< 0.05) higher in the PCOS than in the control group (Table 1). Similarly, serum levels of T, A, and DHEAS were significantly (*p *< 0.05) higher in the PCOS than in the control group (Figure 1). FAI and SHBG (Figure 2), such as fasting insulin concentrations, HOMA, and GIR (Figure 3) also differed significantly (*p *< 0.05) between groups.

**Table 1:** Main clinical data in cases (PCOS group) and controls (control group) at baseline

	PCOS group (n. 45)	Control group (n. 42)	*P*
**Age (yr)**	27.9 ± 3.6	28.2 ± 4.2	0.759
**BMI (Kg/m^2^)**	24.5 ± 2.7	24.8 ± 3.0	0.607
**WHR**	0.8 ± 0.1	0.7 ± 0.1	0.035
**Ferriman-Gallwey score**	10.1 ± 2.5	5.1 ± 3.6	< 0.001
**OGTT**			
**AUC_glucose _(mg/dL/120 min)**	1012.1 ± 51.6	1061.7 ± 99.4	0.005
**AUC_insulin _(μU/mL/120 min)**	8698.0 ± 2715.9	3898.5 ± 1895.5	< 0.001
**AUC_glucose_/AUC_insulin _ratio**	0.13 ± 0.01	0.25 ± 0.14	< 0.001

At each follow-up assessment, serum T, A and DHEAS levels were significantly (*p *< 0.05) higher in PCOS than in control group (Figure 1). Significant (*p *< 0.05) increases at the 20^th ^and 32^nd ^weeks of gestation were observed in T and DHEAS levels, in the PCOS group alone, whereas they resulted unchanged in the control group. In both PCOS and control groups, A levels resulted unchanged throughout pregnancy (Figure 1).

SHBG levels and FAI were significantly (*p *< 0.05) lower and higher, respectively, in PCOS than in control group (Figure 2). In both PCOS and control groups, SHBG levels were significantly (*p *< 0.05) increased at each follow-up visit, while FAI resulted significantly (*p *< 0.05) reduced from 20^th ^weeks of gestation (Figure 2).

Fasting insulin levels, GIR and HOMA were significantly (*p *< 0.05) different between PCOS and control groups at each follow-up assessment. In PCOS group, fasting insulin levels and HOMA were significantly (*p *< 0.05) increased at the 20^th ^and 32^nd ^weeks of gestation, whereas GIR significantly (*p *< 0.05) reduced at the same follow-ups. Conversely, no significant change during pregnancy was observed in any insulin sensitivity index in the control group.

Table 2 shows the variations in T, A, DHEAS, SHBG and fasting insulin levels, and in HOMA, GIR and FAI in PCOS and control groups. Significant differences (*p *< 0.05) were observed in the variations in T, DHEAS, SHBG and fasting insulin levels, and HOMA, GIR and FAI between PCOS and control groups at each follow-up assessment. No further significant difference was observed between two groups.

**Table 2:** Variations in serum androgen levels and in insulin sensitivity indexes in the general and sub-populations distinguished on the basis of the presence/absence of adverse obstetric outcomes

Group	PCOS group			Control group		
	**Total population**	**Adverse outcome**	**No adverse outcome**	**Total population**	**Adverse outcome**	**No adverse outcome**

**T (ng/mL)**						
*Δ(0-12)*	0.11 ± 0.02*	0.15 ± 0.01°	0.05 ± 0.02	0.03 ± 0.001	0.04 ± 0.003	0.03 ± 0.002
*Δ(0-20)*	0.22 ± 0.01*	0.29 ± 0.02°	0.16 ± 0.01	0.02 ± 0.002	0.03 ± 0.002	0.02 ± 0.004
*Δ(0-32)*	0.31 ± 0.02*	0.36 ± 0.01°	0.24 ± 0.01	0.02 ± 0.001	0.03 ± 0.002	0.02 ± 0.004
**A (ng/mL)**						
*Δ(0-12)*	0.16 ± 0.01	0.17 ± 0.01	0.17 ± 0.02	0.13 ± 0.01	0.16 ± 0.01	0.12 ± 0.04
*Δ(0-20)*	0.44 ± 0.02	0.43 ± 0.02	0.45 ± 0.02	0.22 ± 0.01	0.20 ± 0.04	0.23 ± 0.03
*Δ(0-32)*	0.74 ± 0.01	0.74 ± 0.01	0.74 ± 0.02	0.25 ± 0.01	0.25 ± 0.03	0.24 ± 0.01
**DHEAS (ng/mL)**						
*Δ(0-12)*	32.25 ± 2.51*	35.69 ± 2.1°	28.97 ± 2.8	10.51 ± 2.16	11.01 ± 2.31	9.43 ± 2.77
*Δ(0-20)*	175.11 ± 4.92*	184.63 ± 5.2°	166.87 ± 4.5	9.70 ± 1.97	11.26 ± 2.05	9.04 ± 2.52
*Δ(0-32)*	185.28 ± 6.42*	193.33 ± 6.13°	172.54 ± 6.98	11.34 ± 2.43	12.12 ± 3.06	9.90 ± 2.28
**SHBG (nmol/L)**						
*Δ(0-12)*	10.71 ± 3.15*	7.48 ± 3.51°	14.13 ± 4.24	26.76 ± 8.34	25.43 ± 8.96	27.61 ± 7.56
*Δ(0-20)*	90.36 ± 9.44*	73.55 ± 8.34°	116.45 ± 9.08	141.37 ± 9.21	139.12 ± 12.02	153.11 ± 8.42
*Δ(0-32)*	93.69 ± 8.81*	72.79 ± 7.53°	119.81 ± 8.90	275.18 ± 12.88	268.07 ± 11.32	282.16 ± 13.02
**FAI (%)**						
*Δ(0-12)*	-1.13 ± 0.73*	-0.69 ± 0.34°	-1.54 ± 0.77	-2.53 ± 0.47	-2.47 ± 0.29	-2.59 ± 0.42
*Δ(0-20)*	-0.92 ± 0.51*	-0.12 ± 0.09°	-1.88 ± 0.54	-3.13 ± 0.62	-2.99 ± 0.89	-3.23 ± 0.55
*Δ(0-32)*	-1.01 ± 0.90*	-0.35 ± 0.06°	-1.73 ± 0.49	-3.35 ± 0.51	-3.19 ± 1.01	-3.42 ± 0.47
**Fasting insulin (μU/mL)**						
*Δ(0-12)*	1.93 ± 0.36*	2.10 ± 0.21°	1.24 ± 0.43°	1.02 ± 0.52	1.26 ± 0.73	0.98 ± 0.36
*Δ(0-20)*	2.64 ± 1.13*	2.98 ± 1.05°	2.12 ± 1.02°	1.41 ± 0.24	1.58 ± 0.89	1.14 ± 0.41
*Δ(0-32)*	2.71 ± 1.55*	3.12 ± 1.23°	2.31 ± 1.51°	1.85 ± 0.73	2.22 ± 0.93	1.44 ± 0.67
**GIR (mg/10^-4^U)**						
*Δ(0-12)*	0.34 ± 0.01*	0.22 ± 0.01°	0.41 ± 0.01	0.12 ± 0.03	0.09 ± 0.01	0.18 ± 0.02
*Δ(0-20)*	1.02 ± 0.03*	0.78 ± 0.04°	1.76 ± 0.02	0.13 ± 0.05	0.10 ± 0.01	0.17 ± 0.05
*Δ(0-32)*	1.33 ± 0.06*	1.02 ± 0.02°	1.64 ± 0.01	0.25 ± 0.03	0.20 ± 0.04	0.30 ± 0.01
**HOMA**						
*Δ(0-12)*	0.62 ± 0.01*	0.84 ± 0.02°	0.46 ± 0.01	-0.18 ± 0.04	-0.20 ± 0.05	-0.10 ± 0.03
*Δ(0-20)*	0.91 ± 0.04*	1.02 ± 0.03°	0.86 ± 0.02	-0.27 ± 0.12	-0.37 ± 0.15	-0.18 ± 0.10
*Δ(0-32)*	1.12 ± 0.06*	1.48 ± 0.05°	0.89 ± 0.03	-0.26 ± 0.06	-0.34 ± 0.05	-0.19 ± 0.06

*p < 0.05 *vs*. control group; °*p *< 0.05 *vs*. no adverse outcome.

A total of 13 out of 45 (28.9%) from the PCOS group and 4 out of 42 (9.5%) from the control group (*p *= 0.044) had adverse pregnancy and/or neonatal outcomes. In particular, pre-eclampsia occurred in 6 and 1 subjects from PCOS and control group, respectively; whereas a pregnancy-induced hypertension was diagnosed in 7 and 3 subjects from PCOS and control group, respectively. Significant differences between groups were observed in fetal birth weight (3121.4 ± 762.1 *vs*. 3459.8 ± 673.2 g for PCOS and control groups, respectively; *p *= 0.008), while gestational age at delivery was not significantly different between groups (37.5 ± 2.7 *vs*. 38.9 ± 2.2 wks for PCOS and control groups, respectively; *p *= 0.641).

In Table 2 are shown the variations in T, A, DHEAS, SHBG and fasting glucose and insulin levels, and in HOMA, GIR and FAI in the sub-populations distinguished on the basis of the presence/absence of adverse pregnancy/perinatal outcomes and adjusted for age and BMI. A significant (*p *< 0.05) difference in serum T, DHEAS, SHBG, and fasting insulin levels, and in HOMA, GIR and FAI was observed between patients who had and who had not adverse pregnancy/perinatal outcomes only in PCOS group, whereas any difference was observed in healthy controls (Table 2).

## Discussion

Based on previous results [1,2], PCOS should be considered a heterogeneous disorder related to a higher risk for complicated pregnancy. In particular, the risk for any adverse pregnancy/neonatal outcome seemed to be affected by specific PCOS features, i.e. ovarian dysfunction and biochemical hyperandrogenism, whereas no significant effect was detected for clinical hyperandrogenism and ovarian morphology [3]. However, the dynamic of both androgens and insulin sensitivity indexes during pregnancy and their relationship with complications were not investigated [3].

Considering the study protocol, elevated androgen levels were observed in PCOS patients just during the pre-pregnancy stage. On the other hand, any pre-pregnancy data regarding insulin resistance was not available in our sample, even if it is likely to hypothesize that insulin levels were higher in PCOS subjects than in healthy controls also before pregnancy. Nevertheless, both insulin resistance and hyperandrogenism are clinical features of PCOS before and during pregnancy, and could be implicated in the development of pregnancy complications. The right timing for these processes are not still clarified and should be further investigated.

In the current study, the potential androgens and insulin sensitivity changes throughout pregnancy were studied in PCOS as well as in healthy women. To this regard, we performed a parallel analysis of a larger previously published clinical study [2], reporting data on hyperandrogenemia and insulin resistance available in the first (i.e. at study entry and at 12^th ^weeks of pregnancy), the second (i.e. 20^th ^weeks of pregnancy), and the third (i.e. 32^nd ^weeks of pregnancy) trimester of pregnancy.

As expected considering the original population and other reports [10-14], PCOS patients were more hyperandrogenic and insulin resistant than healthy controls at study entry. These differences were maintained later throughout pregnancy.

Interesting data rose from the study of the dynamic of the androgens and insulin-resistance indexes. In agreement with our results, Sir-Peterman *et al*. [14] previously showed that PCOS women had significantly higher concentrations of serum androgen levels than non-PCOS women. Moreover, similar profile of androgen concentrations and other sexual steroids during pregnancy in PCOS and healthy women was described by the same authors [14] suggesting the ovarian origin of the androgens, although a placental source cannot be totally discarded. On the contrary, in the current study, significant increases in serum T and DHEAS levels were detected only in women affected by PCOS during the second trimester of pregnancy, and such trend was sustained in the late pregnancy.

The consequences for these findings are not still clear. However, controversial data [15-18] are available in literature regarding the pathogenetic implications of hyperandrogenemia during pregnancy on the complications development. In particular, a significant association between circulating maternal T levels and reduced birth weight was previously shown [12,19]. To this regard, meta-analytic data reported a lower birth weight in the most common hyperandrogenic condition, such as PCOS women. In the current study, a significant difference in birth weight was also reported between PCOS and healthy women. Several potential mechanisms could be involved, i.e. maternal energy homeostasis changes, reduction of nutrient transport through placenta, and direct effect of hyperandrogenism on the fetal growth.

Several authors [15,16] reported high androgen levels in women with pre-eclampsia. In fact, androgens could mediate hemodynamic changes underlying pre-eclampsia development by inducing a state of sympathetic hyperactivity and vascular hyperactivity [20]. To this regard, in the current, such as in previous studies [1,2], a higher rate of pre-eclampsia and pregnancy-induced hypertension has been reported in PCOS women.

Notwithstanding the increase in androgen levels observed in the current study, a reduced bioavailability of androgens was found in pregnant PCOS such as in healthy women. In fact, due to the liver induction of SHBG, the FAI is improved as compared to pregnancy start. Thus, other factors, mainly related to insulin resistance impairment, cannot be excluded in generating pregnancy complications in PCOS. In fact, the insulin resistance indexes measured were worsened at the second and third trimester of pregnancy in our sample of non-obese PCOS patients when compared to healthy controls with similar age and BMI.

Insulin resistance could be *per se *sufficient to induce endothelial dysfunction directly or indirectly through multiple pathways, and thus predispose cardiovascular disease and to major pregnancy complications in the third trimester, including pre-eclampsia and IUGR [21]. In particular, even if not always demonstrated [22], several data [23-26] supported the association between insulin resistance and subsequent pre-eclampsia. In particular, two prospective studies [23,24] showed an increased risk of pre-eclampsia in women with impaired insulin resistance during the second trimester of pregnancy. Similarly, more recent data [25,26] remarked an association also between first trimester insulin resistance and subsequent pre-eclampsia. In fact, insulin resistance, in concert with other contributing factors, including hyperandrogenism [27], inflammation [28] and increased weight gain [29], seems to impair the trophoblastic/placental angiogenesis and to be a causative factor for pre-eclampsia development.

Finally, in order to evaluate the effects of the changes in androgens and insulin sensitivity indexes throughout pregnancy on the pregnancy and/or neonatal outcomes, a sub-analysis of our sample according to the presence/absence of complicated pregnancy was performed. Although on a very small sample, our data on PCOS women confirmed previous report on pregnant women without a specific diagnosis of PCOS [15-18]. In fact, PCOS women who had a complicated pregnancy were more hyperandrogenic and insulin resistant throughout pregnancy in comparison with those who had not a pregnancy complications. Moreover, the dynamic during pregnancy of serum T, DHEAS, SHBG, FAI, fasting insulin, GIR and HOMA differed significantly according to the pregnancy outcomes showing an impairment of androgen and free androgen levels and insulin resistance markers. On the other hand, no relevant differences were obtained in our healthy population between subjects who had a worse pregnancy outcome in comparison with those who had not any adverse outcome.

## Conclusions

PCOS patients have impaired changes in serum androgen levels and insulin sensitivity indexes during pregnancy. These alterations could be implicated in pregnancy and neonatal complications frequently observed in women affected by PCOS. Further data deriving from well-powered studies would be necessary in order to confirm the relationship between hormonal and metabolic dynamic during pregnancy and risk of pregnancy complications in women affected by PCOS, and in order to clarify the specific pathogenetic mechanisms.

## List of abbreviations

A: Androstenedione; AUC: Area under curve; BP: Blood pressure; BMI: Body mass index; CRL: Crown-rump length; DHEAS: Dehydroepiandrosterone sulfate; GIR: Fasting glucose-to-insulin ratio; FAI: Free androgen index; HR: Heart rate; HOMA: Homeostasis model of assessment; IRMA: Immunoradiometric assay; IRB: Institutional Review Board; CV: Intra- and inter-assay coefficients of variation; OGTT: Oral glucose tolerance test; PCO: Polycystic ovaries; PCOS: Polycystic ovary syndrome; RIA: Radioimmunoassay; RR: Relative risk; SHBG: Sex-hormone binding globulin; SD: Standard deviation; T: Total testosterone; WHR: Waist-to-hip ratio.

## Competing interests

The authors declare that they have no competing interests.

## Authors' contributions

SP conceived of the study, and participated in its design and coordination. FA conceived of the study, participated in the study design and performed the statistical analysis. MR, TR and AD participated in the patients' enrolment. FO, AT and FZ participated in the manuscript drafting and critical discussion. All authors read and approved the final manuscript.
